# Body Fat Measurements in Singaporean Adults Using Four Methods

**DOI:** 10.3390/nu10030303

**Published:** 2018-03-05

**Authors:** Xinyan Bi, Yi Ting Loo, Christiani Jeyakumar Henry

**Affiliations:** 1 Clinical Nutrition Research Centre (CNRC), Singapore Institute for Clinical Sciences (SICS), Agency for Science, Technology and Research (A*STAR), 30 Medical Drive, Singapore 117609, Singapore; Bi_xinyan@sics.a-star.edu.sg (X.B.); Loo_Yi_Ting@sics.a-star.edu.sg (Y.T.L.); 2 Department of Biochemistry, Yong Loo Lin School of Medicine, National University of Singapore, Singapore 117599, Singapore

**Keywords:** body fat, air-displacement plethysmography, dual-energy X-ray absorptiometry, bioelectrical impedance analysis, skinfold

## Abstract

Few studies have been conducted to measure body composition in Asian populations. In this study, we determined the percent body fat (PBF) by using dual-energy X-ray absorptiometry (DEXA), air-displacement plethysmography (ADP or BOD POD), bioelectrical impedance analysis (BIA) and skinfold (SKF) in 445 healthy Singaporean adults. We observed that the BOD POD, BIA and SKF estimates of PBF were highly correlated with that from DEXA (as a reference method) among Singaporean adults. However, they all underestimated PBF (differences of 3.9% for BOD POD, 5.6% for BIA and 12.5% for SKF). Our results filled a gap in the literature by testing the relationships between DEXA and BOD POD, BIA and SKF in a large sample with a wide range of body mass index (BMI) from 16.1 to 37.5 kg/m^2^ and age from 21 to 69.2 years. The differences of PBF measured by different methods were dependent on age, gender and ethnicity. No significant difference was observed between DEXA and BOD POD in men aged > 40 or in BMI tertile 3. However, the mean difference between DEXA and BOD POD was significant in women. Different measuring methods of estimating PBF therefore must be cautiously interpreted.

## 1. Introduction

Previous studies have suggested that having more body fat is linked to a higher prevalence of chronic diseases, including cardiovascular disease (CVD), hypertension, type 2 diabetes (T2D), dyslipidemia and several cancers [1,2]. Despite similar relations between excess body fat and metabolic disorders in Asian and Western populations, [3] there is substantial evidence indicating fat patterning and percent body fat (PBF) differ between these populations. Asians have a greater PBF at a lower body mass index (BMI) than do Westerners. Since most fundamental body composition studies have been conducted in USA and Europe and most assumptions are based on data obtained from Westerners, it is important to determine whether these methods are valid in tracking body composition changes in Asians. It is therefore of interest to compare several measuring methods used to access PBF in Asians. The accurate measure of PBF is necessary for risk assessment in order to develop preventive strategies among this population.

Dual-energy X-ray absorptiometry (DEXA) is a well-accepted reference method for body composition measurements [4,5,6]. Although DEXA is highly precise, it is expensive and exposes participants to some radiation [5]. Some other techniques, including air-displacement plethysmography (known by the trade name BOD POD) and bioelectrical impedance analysis (BIA), have been developed to quantify human body fatness in controlled laboratory conditions. Compared to DEXA, BOD POD and BIA are less expensive and more easily operated. BOD POD calculates PBF from body density, estimated from body volume and body mass [7]. Previous studies [8,9] have validated BOD POD against hydrostatic weighing (HW), the long-held gold standard method of body composition analysis [10]. Unlike HW, BOD POD is much more feasible and comfortable and able to measure individuals to which HW is not appropriate such as obese, elderly and disabled [8]. Recently, BIA is increasingly being used to assess body composition in clinical and research setting [11,12]. The advantages of BIA include ease of use and its portability, making it suitable for large-scale studies. Although several studies have validated BIA against DEXA [13,14], the validity is affected by gender, age, disease state, level of fatness and ethnic backgrounds [15].

One alternative field technique that is simple and non-invasive is skinfold (SKF) measurement. SKF measures the thickness of two layers of skin and the underlying subcutaneous fat. To standardize SKF measurements, guidelines for the anatomical location of SKF sites and measurement technique have been published [16]. SKF provides good estimates of PBF. Several equations for predicting PBF from SKF and anthropometric measurements have been developed and validated for use in Caucasians, such as those of Durnin and Womersley [17] and Jackson and Pollock [18]. However, there remains disagreement concerning whether these equations are applicable to other ethnic groups [19,20]. Previous study shown that the equations of Durnin and Womersley had low validity for determining PBF in rural Guatemalan adults [21]. Another study indicated that the combination of the equations of Durnin and Womersley with Siri’s equation [22] tended to overestimate body fatness in Chinese adults with high levels of body fat [23].

With the presence of various body composition measurement methods, they all have limitations and some degree of measurement errors. Moreover, population groups can differ in body build and adiposity distribution [24]. For example, Chinese are known to have relative short legs compared to body height [25]. Moreover, the “best” measure of PBF in Asian population remains contentious. In this study, we assessed the PBF of healthy Singaporean adults by four techniques, i.e., DEXA (as a reference method), BOD POD, BIA and SKF. We examined the relationships between them by using a large sample with a wider range of BMI and age.

## 2. Methods

### 2.1. Study Design

This study was a cross-sectional analysis of data from 445 participants attending a baseline visit from 17 June 2014 to 24 January 2017. It is consistent with the STARD (standards for the reporting of diagnostic accuracy studies) reporting guidelines.

Participants were recruited from the general public in Singapore through advertisements on newspaper and posters that were placed around the National University of Singapore campus, public area and on the Clinical Nutrition Research Centre (CNRC) website. To be eligible, participants were required to be Singaporeans or individuals who have lived in Singapore for at least five years, healthy men and women. Participants were excluded if they were pregnant or diagnosed with any major diseases. Prior to the study, all participants were asked to restrict alcohol and caffeine-containing drinks as well as to refrain themselves from intense physical activity within 24 h. All procedures involving human subjects were approved by the National Healthcare Group Domain Specific Review Board (NHG DSRB, Reference Number: 2013/00783), Singapore.

### 2.2. Anthropometry

Anthropometric measurements were obtained in the fasting state. Body weight and height measurements were done in duplicate. Weight (kg) in light clothing without footwear was measured to the nearest 0.1 kg by using an electrical scale and height (cm) was measured using a stadiometer to the nearest millimeter (Seca 763 digital scale, Birmingham, UK). BMI (kg/m^2^) was calculated using weight divided by the height squared. Waist circumference (WC) was taken at the smallest WC above the umbilicus or navel and below the xiphoid process. Hip circumference (HC) was taken at the largest circumference around the buttocks and above the gluteal fold. SKF thickness was measured to the nearest 0.1 mm using Holtain Skinfold Calipers (Holtain Ltd., Crymych, UK) at the following six sites: triceps, subscapular, supraspinale, abdominal, thigh and calf on the right site of the body. Two measurements were taken at each site following standard procedures [16,26] and if a difference of >10% was observed, a third measurement was taken. The mean of the two measurements was used in future analysis. The PBF was calculated using the six-site formula for men [(sum of SKF) × 0.1051 + 2.588] and women [(sum of SKF) × 0.1548 + 3.58] published by international society for the advancement of kinanthropometry [16]. Each technician was trained and cross-validated to be able to obtain SKF values that differed from an experienced trainer by less than 10%.

### 2.3. PBF Measurements

DEXA (QDR 4500A, fan-beam densitometer, Hologic, Waltham, MA, USA) was used for the measurement of whole body composition, including fat mass, lean body mass, bone mineral density and content (BMD and BMC). PBF was calculated by using the manufacture’s software (software version 8.21). To ensure accuracy of the measurement, all metal items were removed from the participants.

BOD POD measurements were done by using Bod Pod^TM®^ Body Composition Tracking System (Cosmed, Rome, Italy). A quality control procedure was performed prior to the testing. The participants wore the recommended form-fitting clothing and were weighted on a calibrated electronic scale. After sitting inside the BOD POD chamber, the participants were asked to remain still and breathe normally during measurement. The mean of two measurements was used as the body volume and the PBF was determined using the manufacture’s software (software version 5.2.0).

BIA measurements were carried out using an 8-electrode BIA device (Tanita BC-418, Tokyo, Japan). After entering the age, gender and height of the participants, they were asked to step on the platform barefooted, ensuring that their clothing was not in contact with the scale. The electrodes connected to the foot pads send a low electrical current through the body. The PBF was displayed, which was calculated from prediction equations provided by the manufacture.

### 2.4. Statistical Analysis

Statistical analysis was performed using the Statistical Package for the Social Sciences (SPSS) version 23. All data are expressed as means ± SD. One-way ANOVA was used for between-group comparisons. Linear regression analysis was used to analyze correlations between PBF measured by DEXA, BODPOD, BIA and SKF, respectively. The level of agreement in PBF measured by DEXA and other techniques was assessed using Bland-Altman plots. Two-sided *p* < 0.05 was considered statistically significant in all cases.

## 3. Results

A total of 445 healthy Singaporean adults (184 men and 261 women) were recruited into the study. The anthropometric measures of the participants, along with their mean PBF values as determined by DEXA, BOD POD, BIA and SKF are summarized in Table 1. There were no differences between men and women in mean ages (*p* = 0.805). Generally, men were taller and heavier and tended to have larger BMI, WC and WHR (all *p* < 0.001); whereas women had greater PBF (*p* < 0.001), irrespective of the measurement techniques. As shown in Table 1, the mean PBF measured by BOD POD, BIA and SKF were 3.9%, 5.5% and 12.6%, respectively, lower than that measured by DEXA in all participants (all *p* < 0.001). In men, the mean PBF measured by BOD POD, BIA and SKF were 2.7%, 5.5% and 12.1%, respectively, lower than that measured by DEXA (all *p* < 0.001). In women, the mean PBF measured by BOD POD, BIA and SKF were 4.7%, 5.8% and 13.0%, respectively, lower than that measured by DEXA (all *p* < 0.001).

**Figure 1:**
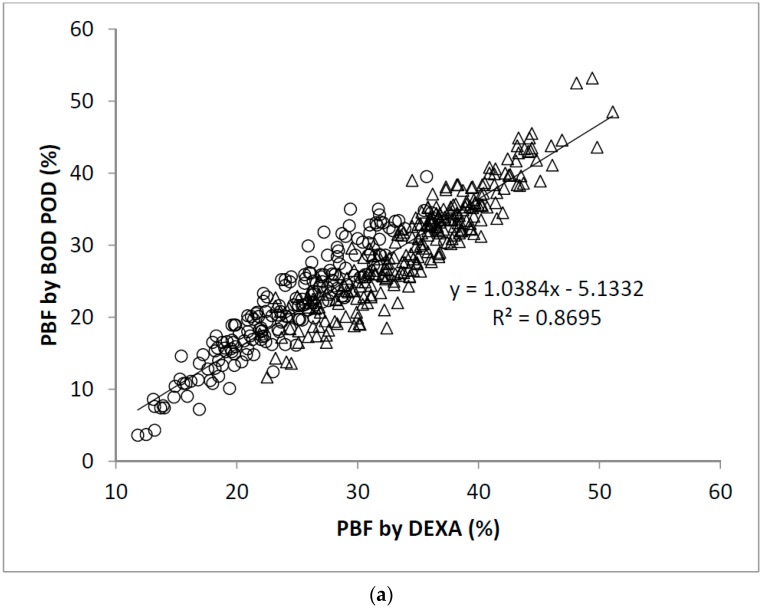

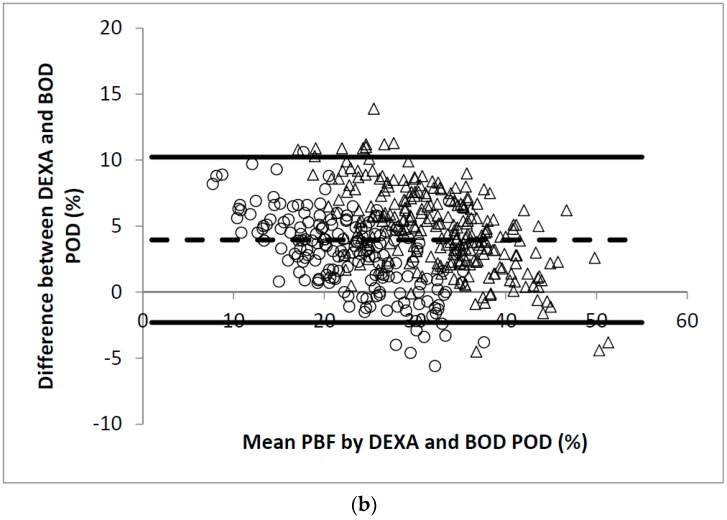
(**a**) Scatter plot of PBF calculated by DEXA and BOD POD in 445 participants. The Pearson’s correlation coefficient is 0.93; (**b**) Bland-Altman plot for data from (**a**). Men were represented by the open circles and women by the open triangles.

**Figure 2:**
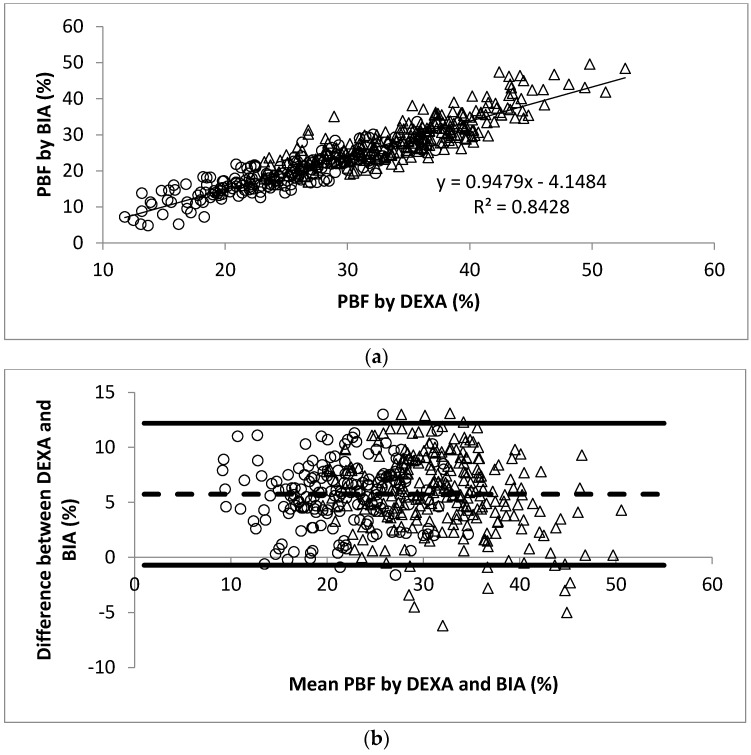
(**a**) Scatter plot of PBF calculated by DEXA and BIA in 445 participants. The Pearson’s correlation coefficient is 0.92; (**b**) Bland-Altman plot for data from (**a**). Men were represented by the open circles and women by the open triangles.

**Figure 3:**
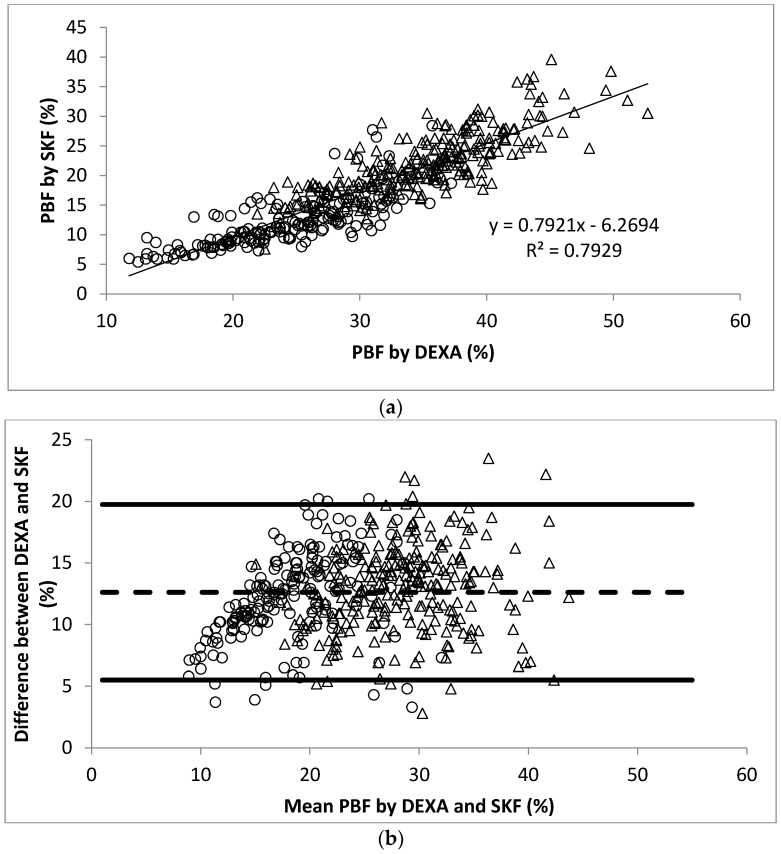
(**a**) Scatter plot of PBF calculated by DEXA and SKF in 445 participants. The Pearson’s correlation coefficient is 0.89; (**b**) Bland-Altman plot for data from (**a**). Men were represented by the open circles and women by the open triangles.

The linear regression analysis showed significant correlation between PBF measured by DEXA and BOD POD (*r* = 0.93, Figure 1a). The Bland-Altman analysis plots of agreement between PBF measured by DEXA and that measured by BOD POD were shown in Figure 1b. The average difference between the PBF measured by DEXA and BOD POD was 4.0% (95% CI −2.3% to 10.2%). Similarly, significant correlation between PBF measured by DEXA and BIA (*r* = 0.92, Figure 2a) and SKF (*r* = 0.89, Figure 3a) were obtained. Figure 2b shows that the average difference between PBF measured by DEXA and BIA was 5.7% (95% CI −0.7% to 12.2%). The Bland-Altman analysis plots of agreement between PBF measured by DEXA and that measured by SKF were shown in Figure 3b. The average difference between the PBF measured by DEXA and SKF was 12.6% (95% CI 5.5% to 19.7%).

Table 2 and Table 3 show that PBF irrespective of the measurement technique, increased across BMI tertiles (all *p* < 0.001). In men, there was no significant difference between DEXA and BOD POD in the top BMI tertile (Table 2). In women, there were no significant differences between BOD POD and BIA in BMI tertiles 1 and 3. When the participants were stratified by age, Table 4 shows that there was no significant difference between DEXA and BOD POD in men with age ≥ 40. However, there was no significant difference between BOD POD and BIA in young women. Age-, ethnicity- and BMI-adjusted partial correlation coefficients are shown in Table 5. In general, the correlations between DEXA, BOD POD, BIA and SKF for PBF measurements were good.

## 4. Discussion

DEXA has been used as a direct technique to measure total and regional body composition [27,28,29]. Prior studies have shown its validity by comparing with other criterion methods [21,22,23,24,25,26,27,28,29,30,31,32]. However, DEXA has some limitations. The radiation dosage, although small, limits its wide applications in certain participants, e.g., pregnancy. Moreover, it is not cost-effective. In most situations, the less expensive and less invasive methods, including BOD POD, BIA and SKF, are the techniques available for body composition measurements.

The results of the present study provide reliable information on the correct interpretation of PBF analyses by BOD POD, BIA and SKF, respectively, using DEXA as the reference method in a large sample with a wide range of BMI and age. We found a significant correlation between BOD POD estimates of PBF and those from DEXA (*r* = 0.93, *p* < 0.001), which is consistent with previous reports [33,34,35,36]. Wingfield et al. [33] found BOD POD to be a valid measure of PBF in overweight and obese women when compared with DEXA, yielding no significant differences. In contrast, Ball et al. [34] reported in 160 male participants a correlation coefficient of 0.94. The mean difference between DEXA and BOD POD was significant (*p* < 0.01) and the difference increased as body fatness increased (*p* < 0.001). Similarly, Radley et al. [35,36] validated that BOD POD estimates of PBF were highly correlated to those derived by DEXA in young adolescents (*r* = 0.84 to 0.95, all *p* < 0.01) as well as in overweight and obese children (*r* = 0.90 to 0.93, all *p* < 0.001). An intriguing finding in our study is that the mean difference between DEXA and BOD POD were altered by age, gender and body fatness (i.e., BMI). No significant differences were observed in men who were in the top BMI tertile (*p* < 0.304) and age > 40 years. However, significant differences were observed in women (all *p* < 0.05). The above mentioned contradictory results from our study and others could be attributed to the differences in participant characteristics, e.g., age, gender, ethnicity.

BIA has become a useful tool for estimation of body composition but some caution must be used in individuals with varied racial backgrounds [37]. It has been reported that the accuracy of BIA to estimate body composition in overweight women [38] or in adolescent girls [39] is affected by the ethnicity. The validity of BIA for estimating PBF with DEXA as a criterion method has been demonstrated in several studies. Sun et al. [40] found discrepancies in PBF measurements by BIA among lean and obese participants when compared with DEXA. BIA tended to overestimate PBF in lean subjects while underestimate PBF in overweight or obese subjects. Similar results were observed in obese children [41]. BIA underestimated PBF and the significant differences between BIA and DEXA were not changed by age and body fatness but by sex. The results from our study confirm that BIA is an alternative for estimating PBF despite the great range of age and BMI of the participants (*r* = 0.92, *p* < 0.001). However, BIA tends to underestimate PBF measured by DEXA in all participants, especially in lean participants (a mean difference of 6.4% for men and 7.6% for women) and old participants (a mean difference of 5.9% for men and 6.6% for women). On the other hand, little variation was observed between BIA and BOD POD measurements in women who were in BMI tertiles 1 and 3 or in young age group. These observations have important implications for the use of BIA to measure PBF in Southeast Asian adults and the interpretation of results. These results, when combined, suggested that BOD POD and BIA can be used to classify Singaporean adults into relative PBF categories at a population level with DEXA as the reference but some caution must be used in participants with low mean BMI.

SKF provides a good estimate of PBF [16] and is suitable for epidemiological measurements. Previous study by Durnin and Womersley [17] was regarded as the seminal study which proposed the idea of using simple, generalized SKF equations to predict PBF. These equations are perhaps the most widely adopted method to estimate PBF in adults. The study by Eston et al. [42] demonstrated the validity of the sum of four upper body SKFs, including biceps, triceps, subscapular and iliac crest, to predict PBF estimated from DEXA. It also confirmed that the combination of lower limb SKFs—i.e., thigh and calf—with the four upper body SKFs provided a significantly more accurate estimation of PBF in healthy young men and women. In the current study, we included supraspinale and abdominal, instead of biceps and iliac crest, in combination with triceps, subscapular, thigh and calf to predict PBF, because Asians have greater total abdominal and intra-abdominal adipose tissue than Caucasian populations [43]. Although we observed a strong correlation between simple SKF measures of PBF and DEXA in the present study (*r* = 0.89, *p* < 0.001), SKF underestimates PBF by more than 12%. It is probably because the six-site prediction equation is not suitable for the present participant. After we completed this study, we found that two skinfold measurements (i.e., biceps and triceps), age, height and WC could be used to predict PBF in this population [44]. Our equations produced a modest deviation in PBF estimation of 1.3 ± 2.9% in the total sample (0.7 ± 3.1% in women and 1.8 ± 2.7% in men).

Several limitations of this study are that while DEXA is considered the gold standard for body composition assessment, some may argue that some factors, e.g., body thickness and hydration may lead to the erroneous results [45] The use of DEXA in Asian populations that have been shown to differ in body composition from Caucasians is needed for further examination. The study also uses a two component (2C) model, which may introduce some error.

## 5. Conclusions

BOD POD and BIA estimates of PBF are highly correlated with that from DEXA among Singaporean adults but they both underestimate PBF (3.9% for BOD POD and 5.6% for BIA). Although SKF is also correlated with DEXA measurement, it has severe limitations in the current population (underestimate PBF by 12.5%). When stratified according to BMI and age, the mean differences of PBF measured by different methods were changed. Therefore, comparisons of PBF among individuals across studies where different assessment methods of PBF are used should be interpreted with caution.

## Figures and Tables

**Table 1:** Characteristics of the study population.

	Total (*n* = 445)	Men (*n* = 184)	Women (*n* = 261)
Age (years)	37.5 ± 14.5	37.3 ± 14.3	37.7 ± 14.7
Ethnicity			
Chinese (*n*)	406 (91%)	162 (88%)	244 (93%)
Non-Chinese (*n*)	39 (9%)	22 (12%)	17 (7%)
Height (cm)	164.5 ± 8.4	171.4 ± 6.0	159.7 ± 6.0 **
Weight (kg)	61.0 ± 12.0	68.5 ± 10.3	55.7 ± 10.2 **
BMI (kg/m^2^)	22.4 ± 3.5	23.3 ± 3.2	21.8 ± 3.5 **
WC (cm)	73.6 ± 9.4	78.6 ± 8.5	70.0 ± 8.3 **
WHR	0.80 ± 0.07	0.85 ± 0.05	0.76 ± 0.05 **
DEXA PBF (%)	30.6 ± 7.8	24.2 ± 5.6	35.1 ± 5.9 **
BOD POD PBF (%)	26.7 ± 8.7	21.5 ± 7.5	30.4 ± 7.5 **
BIA PBF (%)	24.9 ± 8.1	18.7 ± 5.4	29.3 ± 6.6 **
SKF PBF (%)	18.0 ± 7.0	12.1 ± 4.2	22.1 ± 5.4 **

Values are mean ± SD. ** *p* < 0.001 compared to men (one-way ANOVA).

**Table 2:** Descriptive statistics of PBF separated by BMI tertiles for men.

	Tertile 1 (*n* = 64) BMI ≤ 21.6 kg/m^2^	Tertile 2 (*n* = 60) 21.6 < BMI ≤ 24.5 kg/m^2^	Tertile 3 (*n* = 60) 24.5 < BMI ≤ 37.5 kg/m^2^
DEXA	20.6 ± 4.6	24.6 ± 3.7	27.7 ± 5.8 ^a^
BOD POD	16.6 ± 6.0	21.7 ± 5.3	26.5 ± 7.4 ^a^
BIA	14.2 ± 4.0	18.5 ± 3.2	23.6 ± 4.2
SKF	9.9 ± 3.2	11.9 ± 2.9	14.6 ± 4.9

^a^ Indicates no significant difference between DEXA and BOD POD in participants who were in BMI tertile 3 (*p* = 0.304).

**Table 3:** Descriptive statistics of PBF separated by BMI tertiles for women.

	Tertile 1 (*n* = 89) BMI ≤ 20.0 kg/m^2^	Tertile 2 (*n* = 86) 20.0 < BMI ≤ 22.4 kg/m^2^	Tertile 3 (*n* = 86) 22.4 < BMI ≤ 34.3 kg/m^2^
DEXA	30.8 ± 4.5	34.7 ± 4.4	39.8 ± 4.9
BOD POD	24.6 ± 5.8 ^a^	30.2 ± 4.7	36.6 ± 6.5 ^b^
BIA	23.2 ± 3.3 ^a^	28.5 ± 2.7	36.4 ± 5.0 ^b^
SKF	18.2 ± 3.8	21.7 ± 3.7	26.5 ± 4.8

^a^ Indicates no significant difference between BOD POD and BIA in participants who were in BMI tertile 1 (*p* = 0.057); ^b^ Indicates no significant difference between BOD POD and BIA in participants who were in BMI tertile 3 (*p* = 0.769).

**Table 4:** Descriptive statistics of PBF separated by age.

Age (years)	Men		Women	
	21 < Age < 40 (*n* = 113)	40 ≤ Age < 70 (*n* = 71)	21 < Age < 40 (*n* = 156)	40 ≤ Age < 70 (*n* = 105)
DEXA	22.9 ± 5.8 **	26.3 ± 4.6 ^a^	33.1 ± 5.7 **	38.0 ± 4.7
BOD POD	19.4 ± 7.3 **	24.9 ± 6.4 ^a^	27.7 ± 7.0 **^,b^	34.4 ± 6.5
BIA	17.5 ± 5.4 **	20.4 ± 4.9	27.9 ± 6.5 **^,b^	31.4 ± 6.2
SKF	11.6 ± 4.4	12.8 ± 3.9	21.1 ± 5.4 **	23.6 ± 4.9

^a^ Indicates no significant difference between DEXA and BOD POD in men with age ≥ 40 (*p* = 0.339); ^b^ Indicates no significant difference between BOD POD and BIA in women with age < 40 (*p* = 0.826); ** *p* < 0.001 when compared to participants with age ≥ 40.

**Table 5:** Age-, ethnicity- and BMI-adjusted partial correlation coefficients of PBF measured by DEXA, BODPOD, BIA and SKF, respectively, separated by gender.

	Total (*n* = 445)	Men (*n* = 184)	Women (*n* = 261)
DEXA vs. BOD POD	0.93	0.86	0.82
DEXA vs. BIA	0.93	0.76	0.75
DEXA vs. SKF	0.88	0.62	0.59

All coefficients were *p* < 0.001.
